# Spatial proteomics identifies a spectrum of immune dysregulation in acquired bone marrow failure syndromes

**DOI:** 10.3389/fimmu.2023.1213560

**Published:** 2023-09-25

**Authors:** Rachel M. Koldej, Ashvind Prabahran, Chin Wee Tan, Mandy Ludford-Menting, Huw Morgan, Nicholas Holzwart, Melissa J. Davis, David S. Ritchie

**Affiliations:** ^1^ Australian Cancer Research Foundation (ACRF) Translational Research Laboratory, Royal Melbourne Hospital, Melbourne, VIC, Australia; ^2^ Department of Medicine, Faculty of Medicine, Dentistry and Health Sciences, University of Melbourne, Melbourne, VIC, Australia; ^3^ Clinical Haematology, Peter MacCallum Cancer Centre and Royal Melbourne Hospital, Melbourne, VIC, Australia; ^4^ Bioinformatics Division, The Walter and Eliza Hall Institute of Medical Research, Melbourne, VIC, Australia; ^5^ Department of Medical Biology, Faculty of Medicine, Dentistry and Health Sciences, University of Melbourne, Melbourne, VIC, Australia; ^6^ Department of Clinical Pathology, Faculty of Medicine, Dentistry and Health Sciences, University of Melbourne, Melbourne, VIC, Australia

**Keywords:** aplastic anaemia, poor graft function, stem cell transplant, spatial proteomics, bone marrow, microenvironment, autoimmune, inflammation

## Abstract

Poor graft function (PGF), manifested by multilineage cytopenias and complete donor chimerism post-allogeneic stem cell transplantation (alloSCT), and acquired aplastic anaemia (AA) are immune-mediated acquired bone marrow (BM) failure syndromes with a similar clinical presentation. In this study, we used spatial proteomics to compare the immunobiology of the BM microenvironment and identify common mechanisms of immune dysregulation under these conditions. Archival BM trephines from patients exhibited downregulation of the immunoregulatory protein VISTA and the M2 macrophage marker and suppressor of T-cell activation ARG1 with increased expression of the immune checkpoint B7-H3 compared to normal controls. Increased CD163 and CD14 expression suggested monocyte/macrophage skewing, which, combined with dysregulation of STING and VISTA, is indicative of an environment of reduced immunoregulation resulting in the profound suppression of hematopoiesis in these two conditions. There were no changes in the immune microenvironment between paired diagnostic AA and secondary MDS/AML samples suggesting that leukaemic clones develop in the impaired immune microenvironment of AA without the need for further alterations. Of the eight proteins with dysregulated expression shared by diagnostic AA and PGF, the diagnostic AA samples had a greater fold change in expression than PGF, suggesting that these diseases represent a spectrum of immune dysregulation. Unexpectedly, analysis of samples from patients with good graft function post-alloSCT demonstrated significant changes in the immune microenvironment compared to normal controls, with downregulation of CD44, STING, VISTA, and ARG1, suggesting that recovery of multilineage haematopoiesis post-alloSCT does not reflect recovery of immune function and may prime patients for the development of PGF upon further inflammatory insult. The demonstrable similarities in the immunopathology of AA and PGF will allow the design of clinical interventions that include both patient cohorts to accelerate therapeutic discovery and translation.

## Introduction

Acquired aplastic anaemia (AA) and poor graft function (PGF) following allogeneic stem cell transplantation (alloSCT) are acquired bone marrow failure syndromes (BMFS) that lead to infection and bleeding as a result of persisting leukopenia and thrombocytopenia.

Aplastic anaemia has an incidence of 2.35 cases per million people, and mortality rates in newly diagnosed AA are up to 50% and in relapsed AA are up to 70% (1). First-line therapy for patients with AA is dependent on patient age and alloSCT donor availability. Patients who are young and have an appropriate donor are treated using alloSCT. Patients over 40 are at increased risk of transplant-related mortality and are therefore initially treated with immunosuppressive therapies such as antithymocyte globulin + cyclosporin, to which 20% of patients have a poor response and one-third relapse within 2 years (2). Up to 15% of patients will develop secondary myelodysplasia (MDS) or acute myeloid leukaemia (AML) (3–6), which has a poorer prognosis than primary MDS/AML (7).

Poor graft function is a syndrome of severe, life-threatening peripheral cytopenias despite >95% donor engraftment (8). Analysis of our transplant centre data (929 patients, alloSCT 2000–2016) has shown that PGF occurs in 13% of patients undergoing alloSCT, with a mortality rate of 60% in those without bone marrow (BM) recovery (9). Risk factors for PGF include an antecedent diagnosis of myeloproliferative disorder (e.g., myelofibrosis), age ≥50 years, concurrent graft vs. host disease (GVHD), pre-engraftment infection, early ICU admission, and post-engraftment viral infection (9). There are currently no standardised treatments for patients with PGF. Most patients are treated with supportive care (monitoring of blood counts, transfusions, and G-CSF as required), though the use of repeat stem cell infusions, thrombopoietin (TPO) agonists, reactive oxygen species scavengers, and treatment with donor-derived mesenchymal stem cells has been reported (10). The immunopathology of PGF has not been previously described.

Aplastic anaemia is a condition of hyperactive T-cell activation resulting in excessive interferon-γ (IFN-γ) production, which in turn suppresses HSC proliferation, inducing T-cell-mediated HSC apoptosis and the clinical presentation of pancytopenia (11). In addition, mouse models of acquired bone marrow failure have demonstrated that IFN-γ induces tumour necrosis factor alpha (TNF-α) (12) and CCR5 expression (13) in BM resident macrophages, inducing further IFN-γ expression and setting up a positive feedback loop of IFN-γ/TNF-α production and a hyper-inflammatory state. Similarly, excessive IFN-γ has been demonstrated in mouse models of GVHD-driven BM aplasia (14). Analysis of primary AA patient samples has demonstrated skewing of CD4 and CD8 T-cell memory subsets (15, 16), reductions in T regulatory cells (17), oligoclonal expansions of CD8 T cells (18), including loss or mutation of HLA (19, 20), and increased IFN-γ expression in T cell and monocyte subsets (12, 15). However, few studies have examined the immunology of PGF in primary patient samples or the co-contribution of dysregulated myeloid and lymphoid lineages within the BM immune microenvironment of AA.

The similar clinical presentations and potentially IFN-γ driven inflammatory pathology described by others lead us to hypothesise that AA and PGF share similarities in the mechanism of disease pathogenesis and immunobiology. In this study, we utilised primary patient BM samples to directly compare the immunopathology of patients with PGF and AA.

## Methods

### Patient cohort

The analysis of archival samples left over from diagnostic procedures was approved under a waiver of consent by the Melbourne Health Human Research Ethics Committee (Project 2018.239) and conducted in accordance with the Declaration of Helsinki. Clinical data were obtained from review of patient records (**Supplementary Table S1**).

Peripheral blood samples were collected with informed consent from patients at the Peter MacCallum Cancer Centre and The Royal Melbourne Hospital (Melbourne, Australia) under ethics approval of the Melbourne Health Human Research Ethics Committee (Projects 2016.207, 2018.017, and 2019.280) and in accordance with the Declaration of Helsinki. Peripheral blood mononuclear cells (PBMC) were isolated using Ficoll–Paque Plus (GE Healthcare, Chicago, IL, USA) density gradient separation and cryopreserved until required. Peripheral blood samples from age-matched healthy donors were obtained from the Australian Red Cross Blood Service with ethics approval from the Melbourne Health Human Research Ethics Committee (Project 2013.288).

Through a review of our centre records, patients with appropriate BM trephine samples available were selected. Patient characteristics are listed in **Supplementary Table S1**. For severe AA, patients with diagnostic and progression to myeloid malignancy samples prior to alloSCT were selected, of whom six patients had paired diagnostic and progression samples. Normal controls (NC) were selected from patients undergoing staging for high-grade lymphomas with morphologic and immunophenotypically uninvolved bone marrow biopsies.

For PGF, patients were selected based on the following parameters (1): complete myeloid chimerism at the last reading (2), Hb ≤85, neutrophils ≤1.0 × 109/L (3), platelets ≤100 × 109/L for 30 days post-D30. Morphological disease must not be present. As a control, a cohort of patients with good graft function (GGF) post-alloSCT was selected if they had normal blood counts, complete donor chimerism, and no features of disease relapse. PGF and GGF patients were matched 1:1 by the following variables: age, sex, disease/disease risk index, conditioning intensity, donor relation, graft, and donor sex match. Trephines from PGF and GGF were taken at a similar time point post-alloSCT ± 10 days. Where possible, a GGF control with identical matching variables to the PGF patients was selected. If this was not possible, patients were matched with a control that had identical age, conditioning intensity, and disease risk index. The final judgement to use control based on the available matched variables was made by two clinical haematologists.

### Spatial proteomics

At the time of sample collection, BM trephines were processed using standard diagnostic laboratory practice (fixation in B5, decalcification in acid, and paraffin embedding). From identified archival BM trephine blocks, 4 μm sections were cut and mounted on super-frosted slides. Spatial proteomics was performed using the GeoMX platform as previously described (21). Briefly, the tissue area of interest was located using fluorescence imaging, and 6 × 300 µm regions of interest were selected by dual CD45+/CD3+ expression for each trephine sample (**Supplementary Figure S1**). For multiplexed protein expression, samples were analysed using the GeoMX Immune Cell Profiling Core, IO Drug Target Module, Immune Activation Status Module, Immune Cell Typing Module, and Pan-Tumour Module to determine the expression of 57 proteins (**Supplementary Table S2**). As this analysis used predesigned panels, it included markers that are not known to be expressed in the BM, such as MART1, Her2, and NY-ESO-1. These markers were included in the statistical analysis but were not considered further for the dissection of tissue pathology.

### Flow cytometry analysis

Details of the antibodies used are listed in **Supplementary Table S3**. PBMC were stained with Live/Dead Aqua (Thermo Fisher, Waltham, MA, USA) for 30 min at 4°C, washed in FACS buffer (2% FBS in PBS), and blocked in Fc block (BD Biosciences, Franklin Lakes, NJ, USA) and CellBlox Blocking Buffer (Thermo Fisher) for 10 min at RT. Cells were stained with NovaRed685-antiCD25 for 30 min at 4°C, followed by remaining surface antibodies for a further 30 min at 4°C. Cells were washed twice in FACS buffer and permeabilised using Cytofix/Cytoperm Kit (BD Biosciences) according to the manufacturer’s instructions. Cells were stained with BV421-antiSTING in Perm/Wash buffer for 30 min at 4°C prior to two washes with perm/wash buffer. Samples were resuspended in FACS buffer, acquired on an Aurora Spectral Flow Cytometer (Cytek Biosciences, Fremont, California, USA), and analysed using FlowJo (BD Biosciences). The representative gating strategy is shown in **Supplementary Figure S2**.

### Immunohistochemistry

Tissue sections of BM trephines were baked for 2 h at 65°C and deparaffinised/rehydrated using a Leica Jung XL Autostainer (Leica Microsystems Pty Ltd, Macquarie Park, Australia). Antigen retrieval was performed using 10 mM of sodium citrate buffer at pH 6 (Chem Supply, Gillman, SA, Australia) in a Prestige Medical Pressure Cooker (Aptum Biologics Ltd., Southampton, UK). Slides were allowed to come to room temperature and blocked in 1% bovine serum albumin (Sigma Aldrich, Darmstadt, Germany) in PBS with 0.3% Triton X-100 (Sigma Aldrich, Darmstadt, Germany) for 1 h at room temperature. Slides were stained overnight at 4°C with human anti-STING antibody [EPR13130] (ab198952, Abcam, Cambridge, UK). Sections were post-fixed with 4% paraformaldehyde for 30 min at room temperature, and nuclei were stained with 4′,6-diamidino-2-phenylindole (DAPI, Sigma Aldrich, Darmstadt, Germany) at 0.1 mg/ml for 5 min at room temperature. Sections were mounted in ProLong glass antifade (Thermo Fisher Scientific, Waltham, MA, USA). Images were captured using the Vectra Polaris Automated Quantitative Pathology Imaging System (Akoya Biosciences, Menlo Park, CA, USA) and analysed using the Phenocart 1.1.0 imaging software (Akoya Biosciences, Menlo Park, CA, USA).

### Statistical analysis

Spatial proteomics analyses were conducted as previously described (22). Data exploration and quality checks were conducted using relative log expression (RLE) plots and principal component analysis (PCA). The raw counts were normalised using the trimmed mean of *M*-values (TMM) method (23) to normalise the raw counts (**Supplementary Figure S3**), and PCA was used to identify the progression effect and graft functions as the factors explaining the variation in the data. Differential expression (DE) analysis was undertaken using R/Bioconductor package limma (v3.48.0) (24). For AA studies, comparisons modelled include “AA DX vs. Normal” and “AA PROG vs. Normal”, with batch as a covariate. For PGF studies comparisons undertaken were “PGF vs. Normal” and “GGF vs. Normal”, with batch as a covariate. For all contrasts, the voom-limma with duplication correlation pipeline (25) was used and the TREAT criteria applied (26) (*p*-value <0.05) to conduct statistical tests and to calculate the t-statistics, log-fold change (logFC), and adjusted *p*-values. *p*-values <0.05 were considered statistically significant. Heatmaps were graphed using log normalised counts (logCPM) of DE genes for samples of interest with the R package pheatmap. For box plots, data are graphed as centre line, median; box limits, first and third quartiles; whiskers, 1.5 × interquartile range; and points, outliers.

Flow cytometry data were analysed in GraphPad Prism Version 6.07 using a Kruskal–Wallis one-way ANOVA with Dunn’s multiple comparisons tests. The data are graphed as individual points and mean ± standard deviation.

## Results

### Spatial proteomics identifies dysregulated BM immunity in patients with AA and PGF

**Figure 1:**
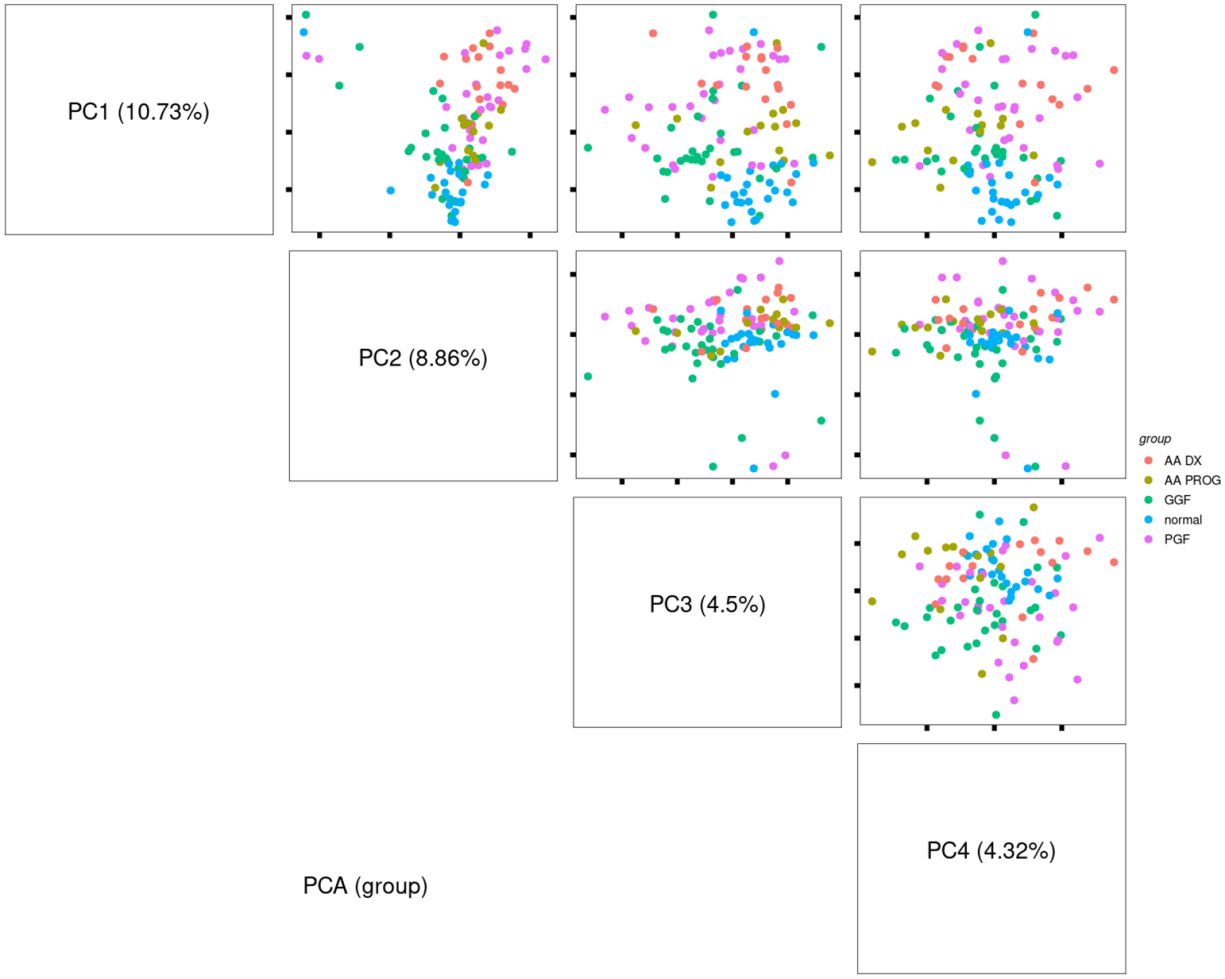
Principal component analysis of study samples demonstrates separation based on disease group along PC1. Comparison of the pattern of protein expression across aplastic anaemia at diagnosis (AA_DX), aplastic anaemia at progression (AA_PROG), poor graft function (PGF), good graft function (GGF), and normal controls (normal).

One of the major barriers to the study of patients with AA or PGF is a lack of cryopreserved BM samples stored at the time of diagnosis that are suitable for immunological examination by either flow cytometry or single-cell gene expression analysis. We have recently described the application of spatial proteomics using the GeoMX platform, which enables the analysis of BM immunity in archival trephine samples (21, 22). This has allowed us to construct a unique cohort of patients (**Supplementary Table S1**) treated at our centre between 2002 and 2020 to provide the first direct comparison of the immunobiology of AA and PGF. Our analysis consisted of severe AA patients at diagnosis (AA_DX, *n* = 15, median age: 45 (21–50)) and patients with newly diagnosed PGF (n = 20, median age: 55 (19–68)). In addition, we collated samples from patients with severe AA who had progressed to AML/MDS (AA_PROG, n = 15, median age: 47 (25–61)) to allow analysis of immune determinants of AA progression, while a cohort of patients with GGF post-alloSCT (n = 20, median age 49 (19–64)), matched for transplant characteristics to those with PGF, were included to allow dissection of the immune impacts of alloSCT vs. those of PGF. The most common indication for alloSCT was AML (45%) and BM trephines were taken at a median of 100 days (60–118) post-alloSCT. Normal controls (n = 20, median age: 51 (18–78)) were included as a reference. Six regions of immune infiltrate per trephine, selected by dual CD45/CD3 staining, were selected (**Supplementary Figure S1**) and analysed for the expression of 57 proteins using predesigned panels to explore immune cell types, memory phenotype, and activation status (**Supplementary Table S2**) to provide a comprehensive overview of the immune microenvironment in each sample. Data were processed and analysed using a spatial proteomics-specific bioinformatics pipeline (22) to control for confounders such as variations in cell number, analysis batch, and intra-patient sampling. For subsequent comparisons, each region of immune infiltrate is reported as a single value, giving six values per sample, which were accounted for in our bioinformatics analysis. All *p*-values are therefore reported as adjusted *p*-values (adj *p*). Analysis of the cohort by PCA showed that normal control patients clustered tightly compared to disease groups. Separation was observed on PC1 based on disease, with AA_DX and PGF samples clustering together away from normal controls (**Figure 1**).

**Figure 2:**
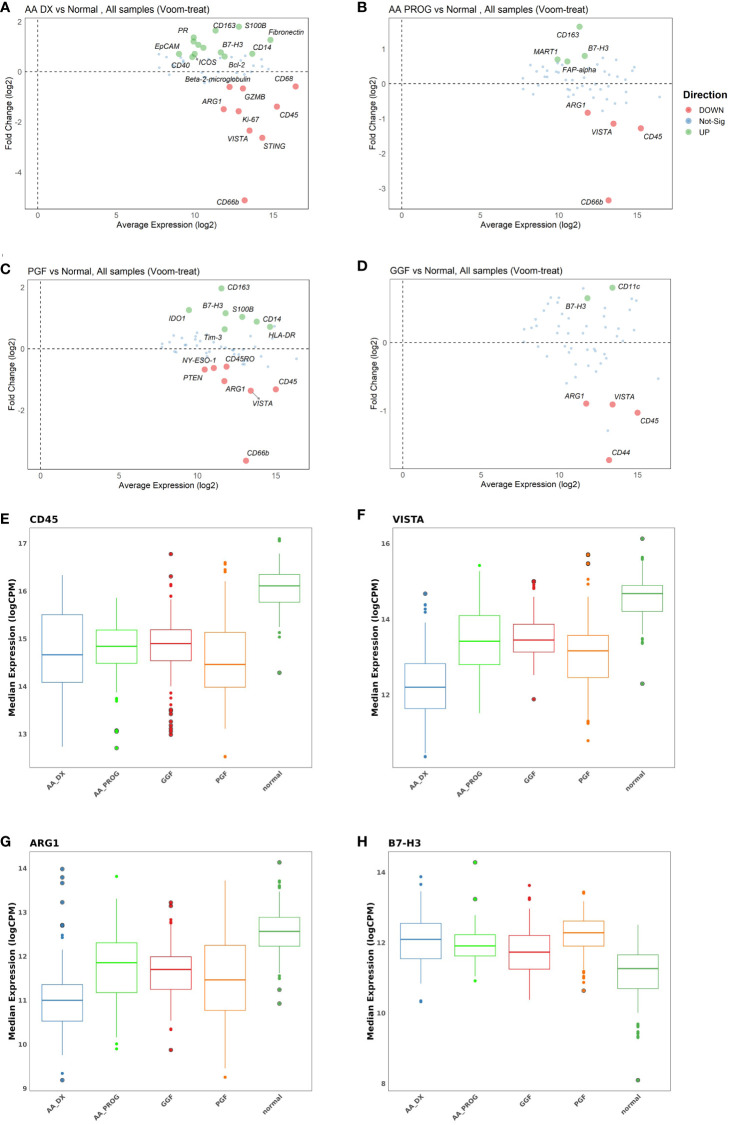
Multivariate analysis identifies significant immune dysregulation across patient groups compared to normal controls. MAplots from multivariate analysis demonstrating changes in expression in **(A)** aplastic anaemia at diagnosis (AA_DX), **(B)** aplastic anaemia at progression (AA_PROG), **(C)** poor graft function (PGF), and **(D)** good graft function (GGF) compared to normal controls. Expression of **(E)** CD45, **(F)** VISTA, **(G)** ARG1, and **(H)** B7-H3 was significantly different across all patient groups compared to normal controls. See **Table 1** for the full list of significant proteins shown in **(A)**.

Compared to normal controls, all patient samples in any of the patient cohorts (AA at diagnosis or progression, PGF or GGF) showed significantly dysregulated immunobiology (**Figures 2A–D**; **Table 1**), with AA_DX having the highest number of significantly differentially expressed proteins. As would be expected given the hypocellular BM of these conditions, expression of CD45 was significantly reduced compared to normal controls across AA_DX (adj *p* = 5.413E−10), AA_PROG (adj *p* = 8.299E−08), and PGF (adj *p* = 8.634E−09) groups (**Figure 2E**; **Table 1**). More surprisingly, patients with GGF showed a similar reduction in CD45 (adj *p* = 1.06E−04). In addition, all patient groups had downregulation of the immunoregulatory protein VISTA and the M2 macrophage marker and suppressor of T-cell activation ARG1 with increased expression of the immune checkpoint B7-H3 compared to normal controls (**Figures 2F–H**; **Table 1**), suggesting a common microenvironment of immune dysregulation leading to marrow hypoplasia.

**Table 1:** Multivariate analysis of significant changes in protein expression vs. normal control.

Comparator	Protein^a^	Log fold change	Average expression	*t*	*p*-value	adj *p*-value
AA_DX	VISTA	−2.342	13.49	−12.18	1.32E−28	6.84E−27
	CD66b	−5.126	13.18	−11.23	3.25E−25	8.46E−24
	STING	−2.635	14.3	−7.831	3.54E−14	6.14E−13
	ARG1	−1.491	11.84	−6.699	4.71E−11	5.41E−10
	CD45	−1.387	15.23	−6.682	5.20E−11	5.41E−10
	PR	1.368	9.935	6.2	8.66E−10	7.02E−09
	S100B	1.796	12.81	6.184	9.45E−10	7.02E−09
	MART1	1.203	9.925	5.337	8.95E−08	5.81E−07
	Her2	1.071	10.23	4.983	5.12E−07	2.96E−06
	Ki-67	−1.576	12.8	−4.869	8.80E−07	4.53E−06
	CD163	1.64	11.32	4.851	9.58E−07	4.53E−06
	FAP-alpha	0.9578	10.54	4.618	2.81E−06	1.22E−05
	Beta-2-microglobulin	−0.6059	12.22	−3.552	2.20E−04	8.80E−04
	GZMB	−0.6705	13.06	−3.375	4.14E−04	0.002
	B7-H3	0.7707	11.65	3.344	4.61E−04	0.002
	ICOS	0.7085	9.991	3.149	8.97E−04	0.002
	Fibronectin	1.268	14.82	3.146	9.05E−04	0.002
	Bcl-2	0.6046	11.9	2.525	0.006	0.017
	CD14	0.7191	13.66	2.495	0.007	0.018
	CD40	0.5862	9.84	2.362	0.009	0.024
	EpCAM	0.7074	8.988	2.359	0.009	0.024
	CD68	−0.5879	16.42	−2.139	0.017	0.039
AA_PROG	CD66b	−3.341	13.18	−7.189	2.30E−12	1.19E−10
	CD45	−1.276	15.23	−5.967	3.19E−09	8.30E−08
	VISTA	−1.145	13.49	−4.882	8.29E−07	1.20E−05
	CD163	1.636	11.32	4.859	9.22E−07	1.20E−05
	B7-H3	0.7994	11.65	3.57	2.06E−04	0.002
	ARG1	−0.8308	11.84	−3.188	7.87E−04	0.007
	FAP-alpha	0.6383	10.54	2.585	0.005	0.038
	MART1	0.6988	9.925	2.525	0.006	0.039
PGF	CD66b	−3.643	13.11	−8.282	4.32E−16	2.24E−14
	CD45	−1.318	15	−6.282	3.32E−10	8.63E−09
	CD163	1.966	11.54	6.103	9.61E-10	1.58E-08
	VISTA	−1.364	13.41	−6.063	1.22E−09	1.58E−08
	B7-H3	1.157	11.8	6.02	1.56E−09	1.62E−08
	ARG1	−1.054	11.72	−4.326	8.96E−06	1.06E−04
	IDO1	1.259	9.463	4.219	1.43E−05	1.40E−04
	CD14	0.885	13.78	3.54	2.17E−04	0.001
	S100B	1.035	12.86	3.05	0.001	0.007
	PTEN	−0.6757	10.46	−2.974	0.002	0.008
	NY-ESO-1	−0.6233	11.05	−2.921	0.002	0.009
	Tim-3	0.6371	11.74	2.31	0.011	0.041
	CD45RO	−0.5795	11.85	−2.299	0.011	0.041
	HLA-DR	0.7142	14.63	2.299	0.011	0.041
GGF	CD45	−1.028	15	−4.526	3.66E−06	1.06E−04
	CD44	−1.72	13.18	−4.503	4.07E−06	1.06E−04
	VISTA	−0.9085	13.41	−3.497	2.53E−04	4.39E−04
	ARG1	−0.8961	11.72	−3.369	4.03E−04	0.005
	CD11c	0.8062	13.4	2.643	0.004	0.041
	B7-H3	0.6522	11.8	2.603	0.005	0.041

a As this analysis used predesigned panels, it included markers that are not known to be expressed in the BM, such as MART1, Her2, and NY-ESO-1. These markers were included in the statistical analysis but were not considered further for the dissection of tissue pathology.

Analysis of the AA_DX cohort reveals changes in both immune cell frequency and function (**Figure 2A**; **Table 1**). Significant downregulation of CD66b (adj *p* = 8.46E−24) is likely reflective of neutropenia in these patients but may also indicate a reduced granulocytic myeloid suppressor cell (MDSC) population. This is augmented by the markedly decreased expression of the immunoregulatory protein VISTA (adj *p* = 6.84E−27), which is highly expressed in immunosuppressive myeloid populations (including MDSCs) but also has a critical role in naïve T-cell maintenance and peripheral tolerance (27). While total myeloid cells were unchanged based on CD11c expression, the combined increase in CD14 (adj *p* = 0.018) and CD163 expression (adj *p* = 4.53E−06) indicated a skewing toward an inflammatory monocyte phenotype. The pathogen recognition and dsDNA sensor protein STING, which plays a central role in both innate and adaptive immunity across dendritic cells and T cells (28), was also decreased in AA_DX patients (adj *p* = 6.14E−13). The decreased expression of both STING and VISTA, combined with increased expression of ICOS (adj *p* = 0.002) and B7-H3 (adj *p* = 0.002), indicates an environment of profound T-cell activation and cytokine production. The nonimmune BM microenvironment is also impacted with increased expression of FAP-alpha (adj *p* = 1.22E−05) and S100B (adj *p* = 7.02E−09), indicating changes in mesenchymal stem cells (29) and Schwann cells (30), respectively. Finally, decreased Ki-67 (adj *p* = 4.53E−06) combined with increased Bcl-2 (adj *p* = 0.017) may reflect an environment of reduced proliferation and apoptosis resistance in residual haematopoietic cells.

### Progression of AA to AML/MDS is not associated with changes to the immune microenvironment

**Figure 3:**
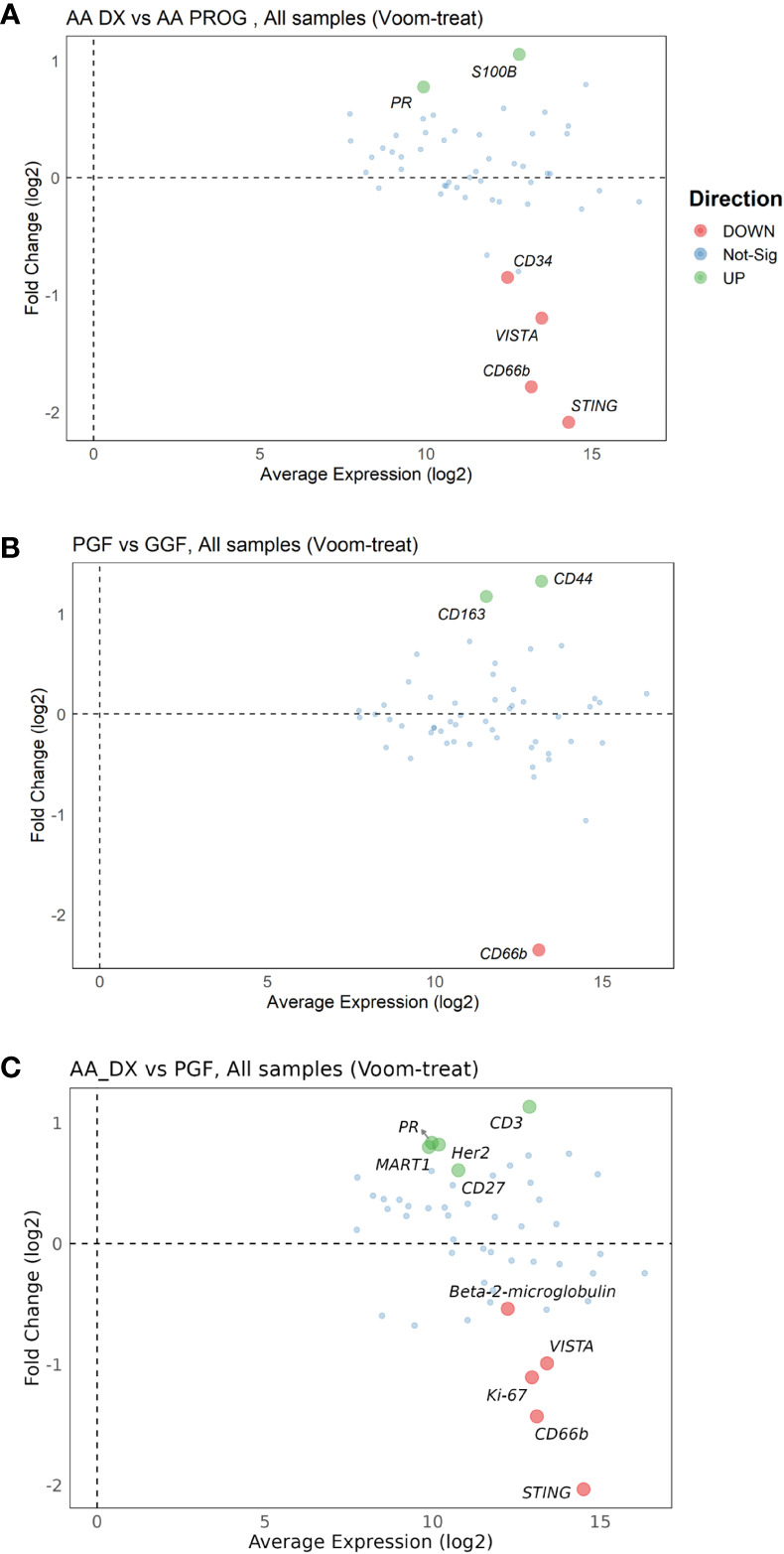
Multivariate analysis of protein expression across AA_DX, AA_PROG, PGF, and GGF. MAplots from multivariate analysis for aplastic anaemia at diagnosis (AA_DX) vs. aplastic anaemia at progression (AA_PROG) **(A)**, poor graft function (PGF) vs. good graft function (GGF) **(B)** and AA_DX vs. PGF **(C)**.

Changes in protein expression between AA_DX and AA_PROG samples were analysed to assess the contribution of the BM immune microenvironment to the progression to myeloid malignancy. When all patient samples were included, STING was the top protein upregulated at progression (adj *p* = 2.59E−06) with CD66b, VISTA, and CD34 also significantly upregulated (**Figure 3A**; **Table 2**). However, when the analysis was restricted to the six patients with matched diagnosis and post-progression samples available, while there was a trend to increased expression of STING, CD34, and VISTA after progression, this was not significant (**Supplementary Figure S4**), indicating that the significant results when all patients are included may be a consequence of inter-patient variation in disease biology rather than disease stage. Furthermore, when the AA_DX samples were separated into those patients who would go on to develop progressive disease vs. those who did not, there were no differentially expressed proteins. Overall, this suggests that the immune microenvironment in AA does not change upon the development of subsequent myeloid malignancies.

**Table 2:** Multivariate analysis of significant changes in protein expression across AA_DX, AA_PROG, PGF, and GGF.

Comparison	Protein^a^	Log fold change	Average expression	*t*	*p*-value	adj *p*-value
AA_DX vs. AA_PROG	STING	−2.088	14.3	−5.452	4.98E−08	2.59E−06
	VISTA	−1.197	13.49	−5.003	4.64E−07	1.21E−05
	CD66b	−1.785	13.18	−3.329	4.92E−04	0.009
	CD34	−0.8491	12.46	−3.198	7.62E−04	0.010
	S100B	1.054	12.81	3.028	0.001	0.014
	PR	0.7754	9.935	2.726	0.003	0.029
PGF vs. GGF	CD66b	−2.355	13.11	−5.139	1.90E−07	9.89E−06
	CD163	1.172	11.54	3.395	3.68E−04	0.007
	CD44	1.328	13.18	3.372	3.99E−04	0.007
AA_DX vs. PGF	STING	−2.033	14.5	−5.413	4.58E−08	2.38E−06
	CD3	1.132	12.88	3.814	7.58E−05	0.001
	VISTA	−0.9908	13.41	−3.8	8.02E−05	0.001
	Her2	0.8201	10.19	3.214	6.91E−04	0.009
	Ki-67	−1.107	12.96	−3.021	0.001	0.012
	PR	0.835	9.977	3.002	0.001	0.012
	MART1	0.7998	9.886	2.73	0.003	0.024
	CD27	0.6077	10.77	2.642	0.004	0.026
	Beta-2-microglobulin	−0.5392	12.24	−2.616	0.005	0.026
	CD66b	−1.43	13.11	−2.592	0.005	0.026

a As this analysis used predesigned panels, it included markers that are not known to be expressed in the BM, such as MART1, Her2, and NY-ESO-1. These markers were included in the statistical analysis but were not considered further for the dissection of tissue pathology.

### Patients undergoing alloSCT have dysregulated BM immunity, which is further dysregulated in PGF

Despite having normal blood counts and full donor chimerism at day 100 post-alloSCT, GGF samples also exhibited a dysregulated BM immune microenvironment. Most significantly, the adhesion marker CD44 was downregulated compared to normal controls (adj *p* = 1.06E−04, **Figure 2D**) and PGF samples (adj *p* = 0.007, **Figure 2B**), with PGF samples exhibiting normal expression of CD44. Expression of VISTA, ARG1, and B7-H3 had a greater degree of dysregulation in PGF patients compared to GGF patients (**Figures 2F–H**), but this difference was not significant (**Figure 3B**), suggesting the ongoing contribution of these proteins to immune dysregulation in the BM post-alloSCT regardless of graft function. Indeed, PCA demonstrated a significant overlap between PGF and GGF samples (**Supplementary Figure S5**).

Within PGF samples, CD163 expression was increased compared to GGF samples (adj P = 0.007), suggesting skewing of monocyte/macrophage populations, with decreased CD66b reflecting the neutropenia of PGF (adj *p* = 9.89E−06) (**Figure 3B**). In addition, multiple proteins were dysregulated in PGF, including upregulation of IDO1 (adj *p* = 1.40E−04), TIM-3 (adj *p* = 0.041), and HLA-DR (adj *p* = 0.041), and downregulation of CD45RO (adj *p* = 0.041) compared to normal controls (**Figure 2C**), suggesting changes in T-cell immunomodulation and/or a bias toward monocytes/macrophages.

### PGF parallels the dysfunctional BM immune microenvironment seen in AA

**Figure 4:**
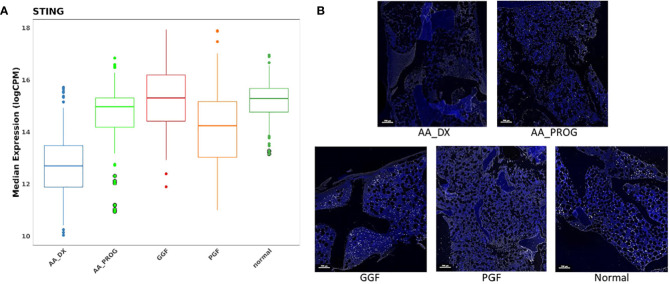
STING is significantly downregulated in the BM of patients with AA_DX and PGF. **(A)** Expression of STING in spatial proteomics multivariate analysis. Refer to **Tables 1** , **2** for statistical analysis. **(B)** Immunohistochemistry of STING expression demonstrating reduced expression in aplastic anaemia at diagnosis (AA_DX) and poor graft function (PGF) (blue = DAPI, white = STING).

PCA of AA_DX and normal controls demonstrated the overlap in the immune microenvironment between AA_DX and PGF, with AA_DX and normal control samples separating into two distinct groups along PC1, with PGF clustering either with AA_DX or between the two groups (**Supplementary Figure S6**). There was greater similarity between AA_DX and PGF samples, with eight of the top 9 proteins dysregulated in PGF also dysregulated in AA_DX samples when compared to normal controls (**Table 1**; **Supplementary Figure S7**), than in PGF and GGF samples, where only CD45, VISTA, ARG1, and B7-H3 are shared (**Figure 2**). However, there were important differences between AA_DX and PGF (**Figure 3C**), with PGF samples having decreased expression of CD3 (adj *p* = 0.001) and CD27 (adj *p* = 0.026), suggesting impaired antigen-specific T-cell responses (31). As outlined above, PGF and AA_DX exhibited a similar pattern of monocyte/macrophage skewing with increased CD163 and CD14 expression. Furthermore, while there were statistically significant differences between these groups for the expression of STING (adj *p* = 2.38E−06), VISTA (adj *p* = 0.001), and CD66b (adj *p* = 0.026), each marker’s expression is highest in normal controls, with decreases in PGF and further decreases in AA_DX samples. This pattern of expression was confirmed by examining STING expression via fluorescent immunohistochemistry (**Figure 4**) in study trephine samples, suggesting that PGF (allogeneic) and AA (autologous) exist on a spectrum of immune dysregulation with a common mechanism of immunopathology in the BM.

### Peripheral blood immunity does not reflect changes in BM immunity

**Figure 5:**
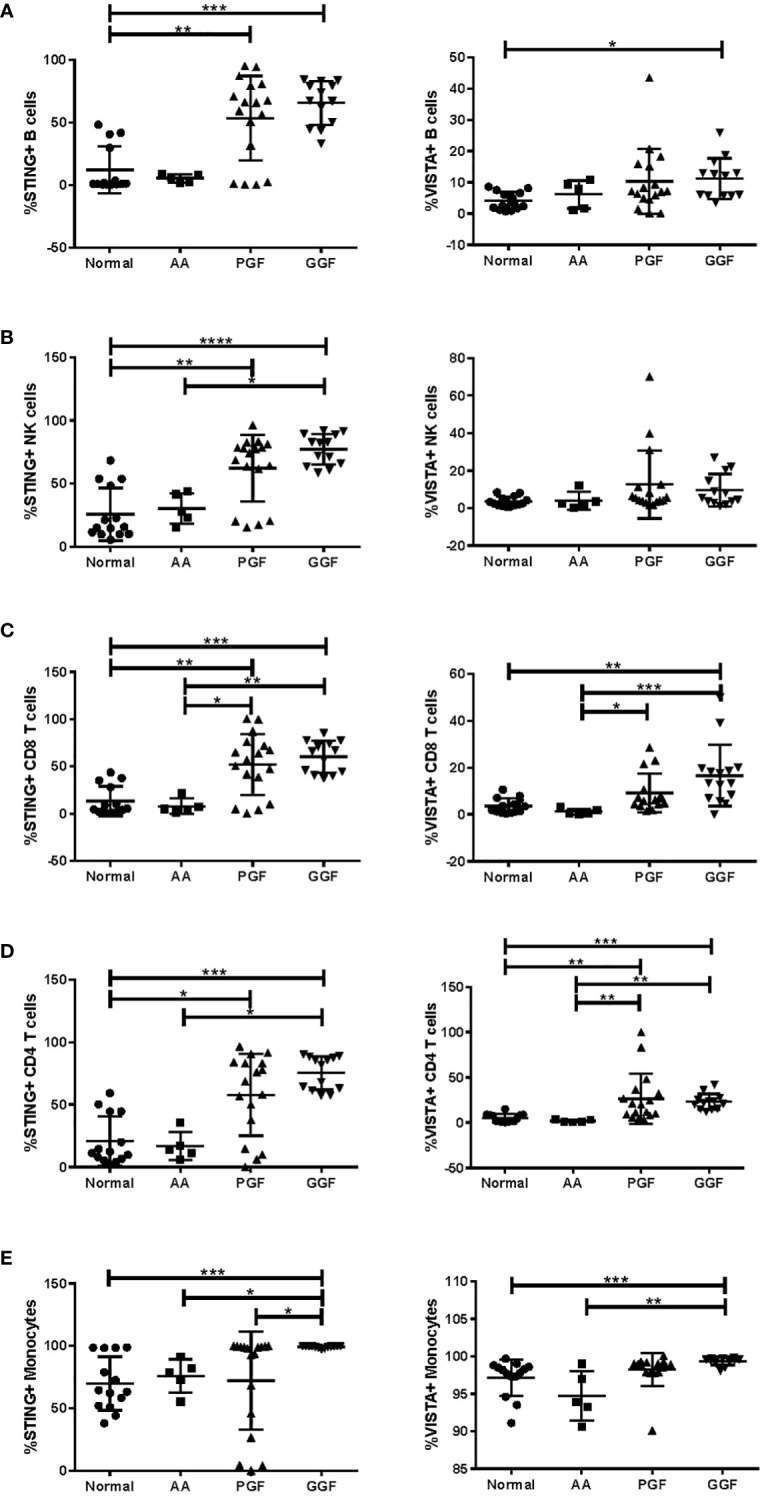
Expression of STING and VISTA in PB immune subsets is significantly different between AA and PGF patients. Flow cytometry analysis of STING and VISTA expression in PB samples from patients with aplastic anaemia (AA) (*n* = 5; median age = 32.5 (range = 27–70); 60% male patients, 40% female patients), poor graft function (PGF) (*n* = 17; median age = 59.0 (range = 40–71); 65% male patients, 35% female patients), good graft function (GGF) (*n* = 13; median age = 59.0 (range = 20–66); 84% male patients, 16% female patients) and normal controls (*n* = 14; median age = 57.0 (range = 25–71); 57% male patients, 43% female patients) across **(A)** B cells, **(B)** NK cells, **(C)** CD8 T cells, **(D)** CD4 T cells, and **(E)** monocytes. ^*^ *p* < 0.05; ^**^ *p* < 0.01; ^***^ *p* < 0.001; ^****^ *p* < 0.0001.

To further confirm the dysregulated expression of VISTA and STING, their expression was examined in PBMC from patients with AA, PGF, and GGF. There were small changes in the proportions of B cells, NK cells, T cells, and monocytes across the groups, with CD4 T cells reduced in patients with PGF and GGF and reduced classical monocytes in AA patients (**Supplementary Figure S8**). Unexpectedly, patients with AA and PGF exhibited significantly different percentages of STING and VISTA-positive cells across B, NK, T, and monocyte subsets (**Figures 5A–E**), with AA patients having expression equivalent to normal controls and most PGF patients having higher expression, equivalent to that of GGF patients. The dysregulated expression was consistent across T-cell memory subsets (**Supplementary Figure S9**). An increased proportion of cells positive for STING and VISTA in patients with GGF provides further evidence of dysregulated immunity in a cohort of patients that are often considered to have normal immunity post-alloSCT. Given the lymphopenia in AA and PGF patients, the absolute number of positive cells is similar, with AA and PGF patients having lower total STING and VISTA positive cells compared to GGF patients (**Supplementary Figure S10**). It should be noted that PBMC samples lack granulocytes, and we were therefore unable to assess changes in STING and VISTA expression in this cell population. Overall, the discordance between peripheral blood (PB) and BM immunity underscores the importance of BM examination in these conditions. Assessment of PB immunology does not necessarily mirror the BM microenvironment and is most likely reflective of ongoing inefficient haematopoiesis, consisting of the cells remaining from disease onset that are not subsequently replaced, rather than the immunopathology of bone marrow failure.

## Discussion

Aplastic anaemia and PGF following alloSCT are acquired BMFS that both present clinically as multilineage cytopenias with BM aplasia. The objective of this study was to investigate the potential of a common BM immunopathology underpinning these conditions. We utilised spatial proteomics analysis of primary archival patient samples from patients treated at our centre over an 18-year period, allowing us to carefully construct our patient cohort to include samples from patients with PGF or GGF and AA patients at diagnosis or post-AML/MDS progression. Our analysis revealed a common microenvironment of immune dysregulation with inflammatory monocyte skewing and increased T-cell activation and identified many potential areas for future investigation for their contribution to BMFS.

Expression of ARG1 was decreased across all patient groups. While commonly used to identify alternatively activated M2 macrophages, ARG1 is also highly expressed in polymorphonuclear neutrophils, where it plays an important role in the suppression of T-cell proliferation and cytokine production (32, 33). Furthermore, the use of ARG1 KO donors in mouse BMT models, when combined with high fat-induced inflammatory conditions (i.e., a Western diet), leads to a decrease in circulating B cells and spleen size (34), suggesting that a reduction in ARG1 expression in GGF patients may prime them for the development of PGF upon a further subsequent injury to the BM such as infection or GVHD. Further reduction in ARG1 in AA and PGF patients is likely the result of ongoing neutropenia and may also contribute to the dysregulated T-cell activation in these conditions.

While this study was primarily designed to examine the immune microenvironment of the BM in these conditions, changes in components of the wider BM microenvironment were detected with increased expression of markers for mesenchymal stromal cells and Schwann cells, suggesting changes to the BM niche and possible effects on HSC quiescence (30). In addition, the T-cell costimulatory protein B7-H3 was upregulated across all patient groups, with the highest expression in PGF patients. Expressed by a wide range of cells, including activated T cells, NK cells, dendritic cells, and macrophages (35–37), along with non-hematopoietic cells, including fibroblasts, synoviocytes, osteoblasts, and epithelial cells (38–40), with both immunostimulatory (41, 42) and inhibitory (43, 44) roles described. Its role in osteoblast differentiation and bone mineralisation (39) suggests that B7-H3 may also be involved in the recovery of the BM post-injury, whether that be chemotherapy-induced or inflammatory-mediated BM aplasia.

Downregulation of both VISTA and STING across patients with AA and PGF is a likely major contributor to immune dysregulation, with VISTA being highly expressed in immunosuppressive MDSCs (45) and a critical regulator of naïve T-cell maintenance (27). In addition, BM-derived macrophages from VISTA KO mice express high levels of CCL3 and CCL5 (46), which have been shown to stimulate CCR5 production in macrophages and IFN-γ production in T cells in mouse models of acquired BMFS (13). The end result is HSC depletion, either directly by IFN-γ increasing the sensitivity of HSCs to T-cell-mediated apoptosis (12, 47) or indirectly by CCR5 increasing inflammatory macrophage persistence and the depletion of CD41+ HSCs (13). While STING classically regulates type 1 IFN responses to dsDNA pathogens and is expressed in most haematopoietic lineages, IFN-independent effects in T cells have been recently described, with STING KO mice exhibiting reduced T-cell death in response to STING agonists (48) and significant effects post-alloSCT, including increased proportions of macrophages and activated dendritic cells and increased CD8 T-cell proliferation and IFN-γ production (49). Additionally, analysis of mouse models of neutropenia has recently demonstrated that IFN-γ signalling in myeloid cells is associated with the functional decline of haematopoiesis (50). Collectively, this suggests that reduced VISTA and/or STING expression results in a dysregulated BM immune microenvironment with reduced immunosuppressive cell populations and increased T-cell activation/proliferation resulting in increased IFN-γ/TNF-α production and HSC depletion. Future investigation of this finding will require complex models that accurately reflect the BM-specific downregulation of both STING and VISTA found in this study to confirm their impact on acquired BMFS.

One intriguing aspect of this study is the dysregulated immunity in patients with GGF post-alloSCT. While the degree of dysregulation was lower than that of patients with PGF, it does suggest that recovery of multilineage haematopoiesis to normal ranges does not necessarily reflect normal immunity, and this dysregulation may impair BM recovery following an inflammatory insult, resulting in PGF. CD44 was specifically downregulated in GGF patients, suggesting possible defects in HSC homing (51) and function (52) and T-cell activation (53, 54) and trafficking to the thymus and lymph nodes (55). In addition, CD44+ CD8 T cells have been shown to mediate anti-tumour responses without inducing GVHD (56), suggesting that downregulation in GGF patients may diminish graft vs. tumour responses. STING has also been examined for its impact on GVHD in both MHC-matched and mismatched mouse models, demonstrating that STING agonists may reduce or prevent GVHD (49, 57, 58). Our analysis is the first to our knowledge to examine STING expression in patient PB and BM samples, demonstrating an increased frequency of STING-positive cells across multiple PB lymphocyte subsets in patients post-alloSCT with STING downregulation in the BM of patients with PGF. Further longitudinal analysis of STING expression in patient samples across the BM, PB, and gastrointestinal tract should be undertaken to understand the dynamics of expression post-alloSCT prior to the application of STING agonists in the clinic.

Our study is the first to describe a common inflammatory immunopathology across AA and PGF in primary patient samples, indicating an environment of reduced immunoregulation and immunosurveillance. There was no difference in the immune microenvironment of AA patients at diagnosis vs. progression to myeloid malignancy, supporting the conclusion by others that secondary MDS/AML is a result of HSC clonal evolution (19, 59, 60) that evades the impaired, yet stable, immune marrow microenvironment. Our results suggest that AA and PGF exist on a spectrum, with AA showing a greater degree of dysregulation. The close monitoring and use of immunosuppression post-alloSCT likely allows for prompt intervention in PGF patients, preventing the degree of dysregulation seen in AA patients, who are only diagnosed when they present with significant persistent cytopenias. The inflammatory trigger for the development of acquired BMFS is unknown, and we are unable to draw any conclusions about this based on our analysis. However, the finding of common immunopathology provides the opportunity to analyse the clinical records of patients with PGF to understand the risk factors for and mechanisms of pathogenesis across acquired BMFS. Furthermore, it will allow for the design of preclinical and clinical studies that include both patient populations, accelerating our understanding of the biology and the development of new treatment strategies.

Recently, TPO mimetics such as eltrombopag have emerged as a new treatment option in AA (61, 62) and post-alloSCT thrombocytopenia (63) with response rates of 44% and 36%, respectively, in prospective studies. TPO mimetics can prevent the induced blockade of endogenous TPO to activate TPO signalling on HSCs and promote HSC survival (64). However, this therapeutic intervention does not interrupt the IFN-γ/TNF-α feedback loop, and its effect on patient immunity is largely unknown. The Janus Kinase 1/2 inhibitor ruxolitinib has recently been shown to reduce T-cell cytokine production in mouse models of immune bone marrow failure (65), and it is soon to be tested in clinical trials, which may provide additional immune-directed therapy for these patients.

This analysis of primary patient BM samples has identified that rather than a single master regulator of immune dysregulation, acquired BMFS presents with multiple mechanisms of immune dysregulation upstream of IFN-γ and TFN-α, all of which likely contribute to the inflammatory BM immunopathology. These require further study and validation in primary patient BM samples and translational mouse models, both in isolation and combination, to determine their relative contribution to acquired BMFS. New therapies for acquired BMFS should be investigated that specifically target the underlying immune dysregulation to reset and recover patient immunity, prevent HSC apoptosis, and lead to improved haematopoietic output and ultimately potential cure of the disease.

## Data availability statement

The raw data supporting the conclusions of this article will be made available by the authors, without undue reservation.

## Ethics statement

The studies involving human participants were reviewed and approved by Melbourne Health Human Research Ethics Committee. The patients/participants provided their written informed consent to participate in this study.

## Author contributions

RK and DR designed and supervised the study. AP sourced patient data and samples. RK, AP, ML-M, HM, and NH performed experiments and analysed data. CT and MD performed bioinformatics analysis. RK wrote the paper with input from other authors. All authors contributed to the article and approved the submitted version.
