# Supplementary material for: Spatial proteomics identifies a spectrum of immune dysregulation in acquired bone marrow failure syndromes
Source: Front Immunol. 2023 Sep 25;14:1213560. doi: 10.3389/fimmu.2023.1213560 (PMC10560754; doi:10.3389/fimmu.2023.1213560)
Supplement: Supplementary file 1 [file DataSheet_1.docx]

**Spatial proteomics identifies a spectrum of immune dysregulation in Acquired Bone Marrow Failure Syndromes**

Rachel M Koldej^1,2*,^ Ashvind Prabahran^1,2,3^, Chin Wee Tan^4,5^, Mandy Ludford-Menting^1,2^, Huw Morgan^1,2^, Nicholas Holzwart^1,2^, Melissa J Davis^4,5,6^ and David S Ritchie^1,2,3^

^1^ACRF Translational Research Laboratory, Royal Melbourne Hospital, Melbourne, VIC, Australia, ^2^Department of Medicine, Faculty of Medicine, Dentistry and Health Sciences, University of Melbourne, Melbourne, VIC, Australia, ^3^Clinical Haematology, Peter MacCallum Cancer Centre and Royal Melbourne Hospital, Melbourne, VIC, Australia, ^4^The Walter and Eliza Hall Institute of Medical Research, Parkville, Melbourne, VIC, Australia, ^5^Department of Medical Biology, Faculty of Medicine, Dentistry and Health Sciences, University of Melbourne, Melbourne, VIC, Australia,^6^Department of Clinical Pathology, Faculty of Medicine, Dentistry and Health Sciences, University of Melbourne, Melbourne, VIC, Australia.

Supplementary Table 1 – Patient details for GeoMX analysis

| **Patient** | **Sex** | **Age at Aplastic Sample** | **Age at MDS Diagnosis** | **Time from diagnosis to progression (years)** |
| --- | --- | --- | --- | --- |
| **Matched Aplastic Anaemia and Progression Samples** | | | | |
| AA1 | M | 16 | 25 | 9 |
| AA2 | F | 38 | 39 | 1 |
| AA3 | F | 42 | 46 | 4 |
| AA4 | M | 60 | 61 | 1 |
| AA5 | M | 48 | 50 | 2 |
| AA6 | M | 50 | 51 | 1 |
|  |  | **Age at Bone Marrow Biopsy** |  |  |
| **Patients with Diagnostic Aplastic Samples only** | | | | |
| AA7 | F | 46 |  |  |
| AA8 | F | 36 |  |  |
| AA9 | F | 64 |  |  |
| AA10 | F | 18 |  |  |
| AA11 | M | 23 |  |  |
| AA12 | M | 57 |  |  |
| AA13 | M | 26 |  |  |
| AA14 | M | 25 |  |  |
| AA15 | M | 48 |  |  |
| AA16 | M | 27 |  |  |
| **Patients with MDS Progression Samples only** | | | | |
| AA17 | M | 56 |  |  |
| AA18 | M | 63 |  |  |
| AA19 | M | 59 |  |  |
| AA20 | F | 26 |  |  |
| AA21 | F | 70 |  |  |
| AA22 | M | 39 |  |  |
| AA23 | F | 34 |  |  |
| AA24 | M | 50 |  |  |
| **Normal Control** | | | | |
| NC1 | M | 30 |  |  |
| NC2 | F | 50 |  |  |
| NC3 | M | 36 |  |  |
| NC4 | M | 54 |  |  |
| NC5 | M | 42 |  |  |
| NC6 | F | 73 |  |  |
| NC7 | F | 45 |  |  |
| NC8 | M | 22 |  |  |
| NC9 | M | 40 |  |  |
| NC10 | M | 88 |  |  |
| NC11 | M | 23 |  |  |
| NC12 | M | 58 |  |  |
| NC13 | M | 78 |  |  |
| NC14 | M | 53 |  |  |
| NC15 | M | 64 |  |  |
| NC16 | M | 61 |  |  |
| NC17 | F | 78 |  |  |
| NC18 | F | 44 |  |  |
| NC19 | F | 77 |  |  |
| NC20 | F | 18 |  |  |

| **Patient ID** | **Sex** | **Age** | **Disease** | **DRI** | **Graft Type** | **Donor Relation** | **HLA Match** | **GVHD** | **Donor(D)/ Recipient (R) sex match** | **Conditioning Intensity** |
| --- | --- | --- | --- | --- | --- | --- | --- | --- | --- | --- |
| PGF1 | F | 39 | CML | Low | BM | sib | HLA-Matched Sibling | Yes | M/F | MAC |
| GGF1 | F | 39 | CML | Low | BM | sib | HLA-Matched Sibling |  | M/F | MAC |
| PGF2 | F | 42 | AML | Int | PB | UD | 12/12 |  | M/F | MAC |
| GGF2 | F | 42 | MDS | Int | PB | UD | 11/12 |  | F/F | MAC |
| PGF3 | M | 55 | Other | Int | PB | sib | HLA-Matched Sibling |  | F/M | MAC |
| GGF3 | M | 51 | AML | Int | PB | UD | 11/12 |  | M/M | MAC |
| PGF4 | F | 25 | SAA | N/A | BM | UD | 11/12 |  | M/F | NMA |
| GGF4 | M | 24 | SAA | N/A | BM | UD | 11/12 |  | M/M | RIC |
| PGF5 | M | 68 | AML | Int | PB | UD | 11/12 |  | M/M | RIC |
| GGF5 | M | 64 | AML | Int | PB | UD | 10/12 |  | M/M | RIC |
| PGF6 | F | 55 | Myelofibrosis | Int | PB | UD | 11/12 | Yes | M/F | RIC |
| GGF6 | M | 55 | AML | Int | PB | UD | 11/12 |  | M/M | RIC |
| PGF7 | M | 61 | MDS | High | BM | UD | 12/12 |  | M/M | RIC |
| GGF7 | F | 59 | AML | High | PB | sib | HLA-Matched Sibling |  | F/F | RIC |
| PGF8 | F | 44 | AML | Int | PB | UD | 12/12 |  | M/F | MAC |
| GGF8 | F | 41 | AML | Int | PB | UD | 11/12 |  | F/F | MAC |
| PGF9 | F | 60 | AML | High | PB | UD | 12/12 |  | M/F | RIC |
| GGF9 | F | 59 | AML | High | PB | sib | HLA-Matched Sibling |  | F/F | RIC |
| PGF10 | F | 57 | Myelofibrosis | Int | PB | sib | HLA-Matched Sibling |  | M/F | RIC |
| GGF10 | M | 57 | Myelofibrosis | Int | PB | sib | HLA-Matched Sibling |  | M/M | RIC |
| PGF11 | F | 31 | AML | High | PB | UD | 12/12 |  | M/F | MAC |
| GGF11 | M | 29 | AML | Int | PB | UD | 10/12 |  | M/M | MAC |
| PGF12 | M | 47 | ALL | Very High | PB | UD | 10/12 | Yes | M/M | MAC |
| GGF12 | F | 47 | ALL | High | PB | UD | 11/12 |  | M/F | MAC |
| PGF13 | M | 19 | ALL | Very High | BM | UD | 10/12 |  | M/M | MAC |
| GGF13 | M | 19 | ALL | High | PB | UD | 12/12 |  | M/M | MAC |
| PGF14 | F | 55 | ALL | High | PB | sib | HLA-Matched Sibling |  | F/F | RIC |
| GGF14 | F | 54 | AML | High | PB | sib | HLA-Matched Sibling |  | F/F | RIC |
| PGF15 | F | 54 | ALL | Int | PB | sib | HLA-Matched Sibling |  | F/F | MAC |
| GGF15 | F | 53 | ALL | Int | PB | sib | HLA-Matched Sibling |  | F/F | MAC |
| PGF16 | F | 55 | Myelofibrosis | Int | PB | UD | 11/12 |  | M/F | RIC |
| GGF16 | M | 55 | AML | Int | PB | UD | 11/12 |  | M/M | RIC |
| PGF17 | F | 23 | AML | High | PB | UD | HLA-Matched Sibling |  | F/F | MAC |
| GGF17 | M | 24 | AML | Very High | PB | UD | 10/12 |  | F/M | MAC |
| PGF18 | M | 20 | ALL | Int | PB | UD | 11/12 |  | F/M | MAC |
| GGF18 | F | 22 | ALL | Int | PB | UD | 11/12 |  | M/F | MAC |
| PGF19 | F | 38 | AML | High | PB | UD | 11/12 |  | M/F | MAC |
| GGF19 | M | 38 | Acute leukaemia of ambiguous lineage | High | PB | UD | 11/12 | Yes | M/M | MAC |
| PGF20 | M | 64 | AML | High | PB | UD | 9/12 (1 x B mm & 2 x DP mm (Permissive) |  | F/M | RIC |
| GGF20 | M | 62 | MDS | High | PB | UD | 12/12 |  | M/M | RIC |

Supplementary Table 2: GeoMX® Digital Spatial Profiling markers (all from NanoString Technologies)

| Module | Proteins |
| --- | --- |
| Immune Cell Profiling Core,  Cat 121300101 | Beta-2-Microglobulin, CD11c, CD20, CD3, CD4, CD45, CD56, CD68, CD8, CTLA4, Fibronectin, HLA-DR, PanCK, PD-1, PD-L1, SMA, GZMB, GAPDH, Histone H3, S6, Ms IgG1, Ms IgG2a, Rb IgG |
| IO Drug Target, Cat 121300102 | Tim-3, 4-1BB, ARG1, B7-H3, GITR, IDO1, LAG3, OX40L, STING, VISTA |
| Immune Activation Status, Cat 121300103 | CD127, CD25, CD27, CD40, CD44, CD80, ICOS, PD-L2 |
| Immune Cell Typing, Cat 121300104 | FOXP3, CD14, CD163, CD34, CD45RO, CD66b, FAP-alpha |
| Pan-Tumour, Cat 121300105 | PTEN, Bcl-2, EpCAM, ER-alpha, Her2, MART1, NY-ESO-1, PR, S100B |

Supplementary Table 3 – Flow cytometry antibodies

| **Laser** | **Specificity** | **Fluorochrome** | **Clone** | **Supplier** | **Cat Number** | **Optimised dilution** |
| --- | --- | --- | --- | --- | --- | --- |
| UV | HLA-ABC | BUV395 | G46-2.6 | BD Biosciences | 565334 | 1/1000 |
|  | Viability | Live/Dead Blue | - | Thermo Fisher | L34966 | 1/2000 |
|  | CD14 | BUV496 | MφP9 | BD Biosciences | 741200 | 1/800 |
|  | CD5 | BUV563 | UCHT2 | BD Biosciences | 741354 | 1/800 |
|  | PD-L1 | BUV661 | MIH1 | BD Biosciences | 741666 | 1/50 |
|  | VISTA | BUV737 | MIH65.rMAb | BD Biosciences | 749648 | 1/50 |
|  | CD4 | BUV805 | SK3 | BD Biosciences | 564910 | 1/200 |
| Violet | STING | BV421 | T3-680 | BD Biosciences | 564966 | 1/50 |
|  | ICOS | SB436 | ISA-3 | Thermo Fisher | 62-9948-42 | 1/50 |
|  | CD11b | Pacific blue | ICRF44 | Biolegend | 301316 | 1/4000 |
|  | CD8 | BV480 | RPA-T8 | BD Biosciences | 566121 | 1/5000 |
|  | CD3 | BV510 | OKT3 | Biolegend | 317331 | 1/800 |
|  | CD16 | BV570 | 3G8 | Biolegend | 302036 | 1/1000 |
|  | CD62L | BV605 | DREG-56 | Biolegend | 304834 | 1/1000 |
|  | TCRgd | BV650 | 11F2 | BD Biosciences | 745359 | 1/400 |
|  | CD163 | BV711 | GHI/61 | BD Biosciences | 563889 | 1/100 |
|  | CD123 | BV750 | 9F5 | BD Biosciences | 747136 | 1/1600 |
|  | HLA-DR | BV786 | G46-6 | BD Biosciences | 564041 | 1/2000 |
| Red | CD19 | APC | SJ25-C1 | BD Biosciences | 340437 | 1/400 |
|  | LAG-3 | Alexa 647 | T47-530 | BD Biosciences | 565716 | 1/50 |
|  | CD25 | NovaRed 685 | BC96 | Phitonex | Custom manufacture | 1/50 |
|  | CD56 | A700 | B159 | BD Biosciences | 557919 | 1/200 |
|  | CD127 | APC-eF780 | eBioRDR5 | Thermo Fisher | 47-1278-42 | 1/100 |
| Blue | PD-1 | BB515 | EH12.1 | BD Biosciences | 564494 | 1/50 |
|  | CD45RA | PerCP-Cy5.5 | HI100 | Thermo Fisher | 45-0458-42 | 1/200 |
|  | CD15 | PerCP e710 | MMA | Thermo Fisher | 46-0158-42 | 1/400 |
| yellow/green | CCR7 | PE | 3D12 | BD Biosciences | 552176 | 1/100 |
|  | TIM3 | PE-eF610 | 7D3 | Thermo Fisher | 61-3109-42 | 1/50 |
|  | CD66b | PE-Fire640 | 6/40c | Biolegend | 392918 | 1/100 |
|  | CD11c | PE-Cy5 | B-ly6 | BD Biosciences | 551077 | 1/800 |
|  | CD33 | PE-Cy7 | P67.6 | BD Biosciences | 333946 | 1/800 |

**
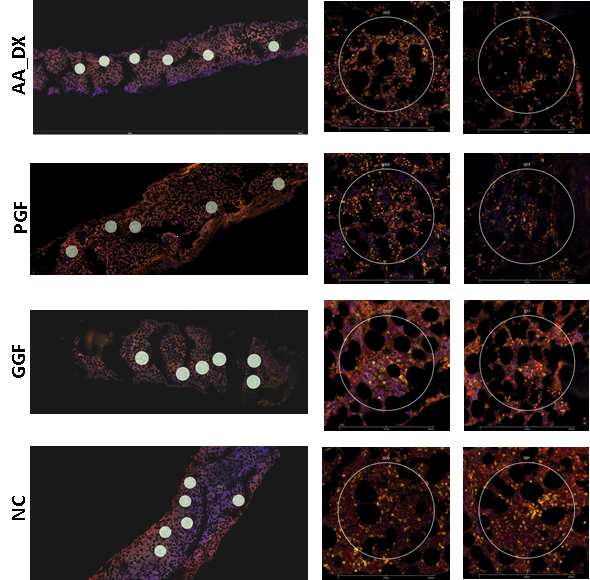
**

**Supplementary Figure 1: Immunoflourescent staining of BM trephine sections for the identification of immune clusters by CD45/CD3 staining.** Left – representative images of the trephines showing selection of regions of interest. Right – representative 300µm regions of interest for subsequent proteomics analysis. Blue – SYTO13 DNA nuclei stain, Green – Alexa 594 anti hCD45 (NanoString Technologies, Cat 121300301), Red – APC anti hCD3 (Miltenyi Biotech, Cat 130-120-269)


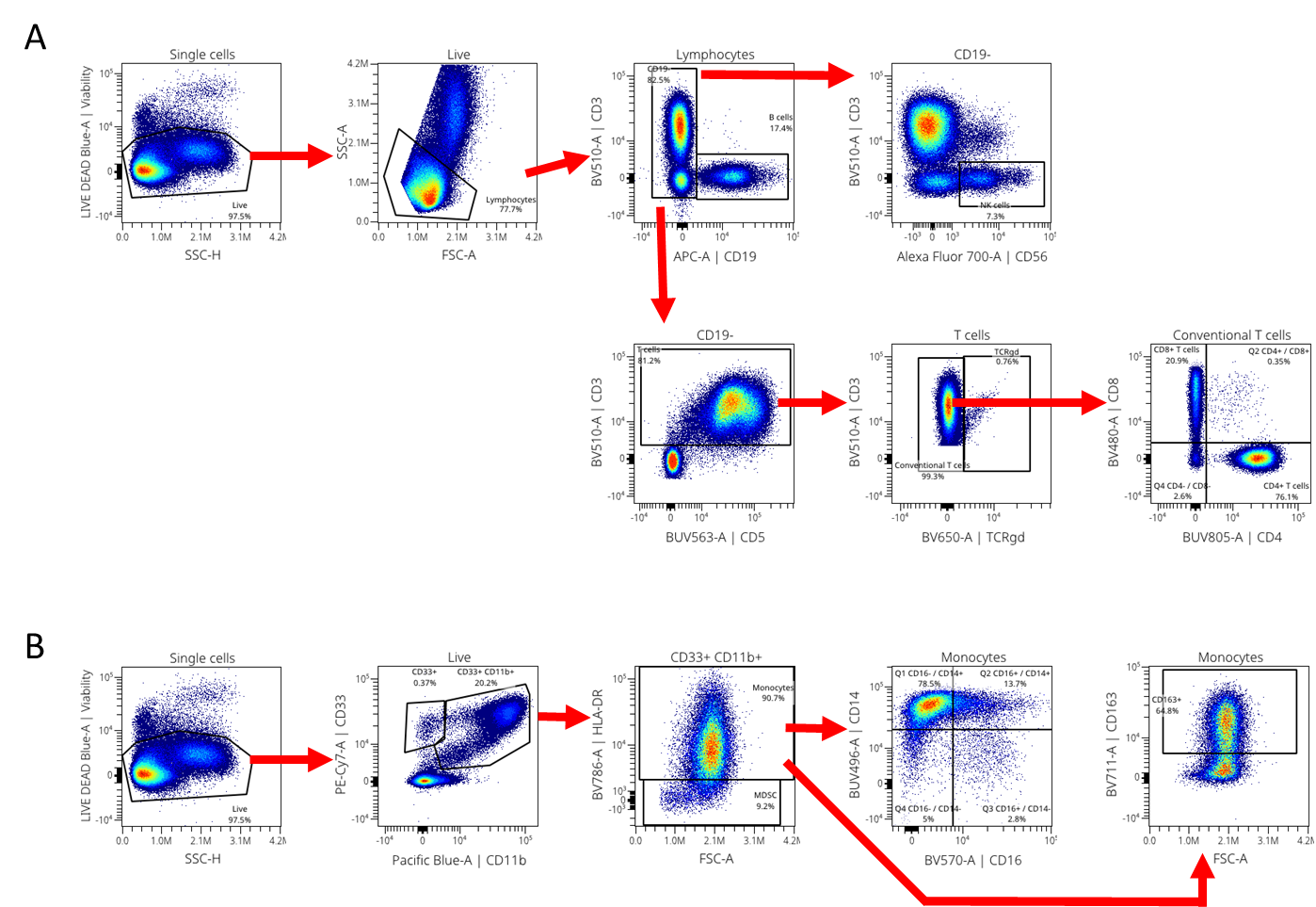


**Supplementary Figure 2 – Flow cytometry gating strategy**. Lymphocyte gating strategy (A) for identification of B cells, NK cells and CD4 and CD8 T cells. Monocyte gating strategy (B) for identification of monocytes and monocyte subsets. Expression of STING and VISTA was determined on each subset.


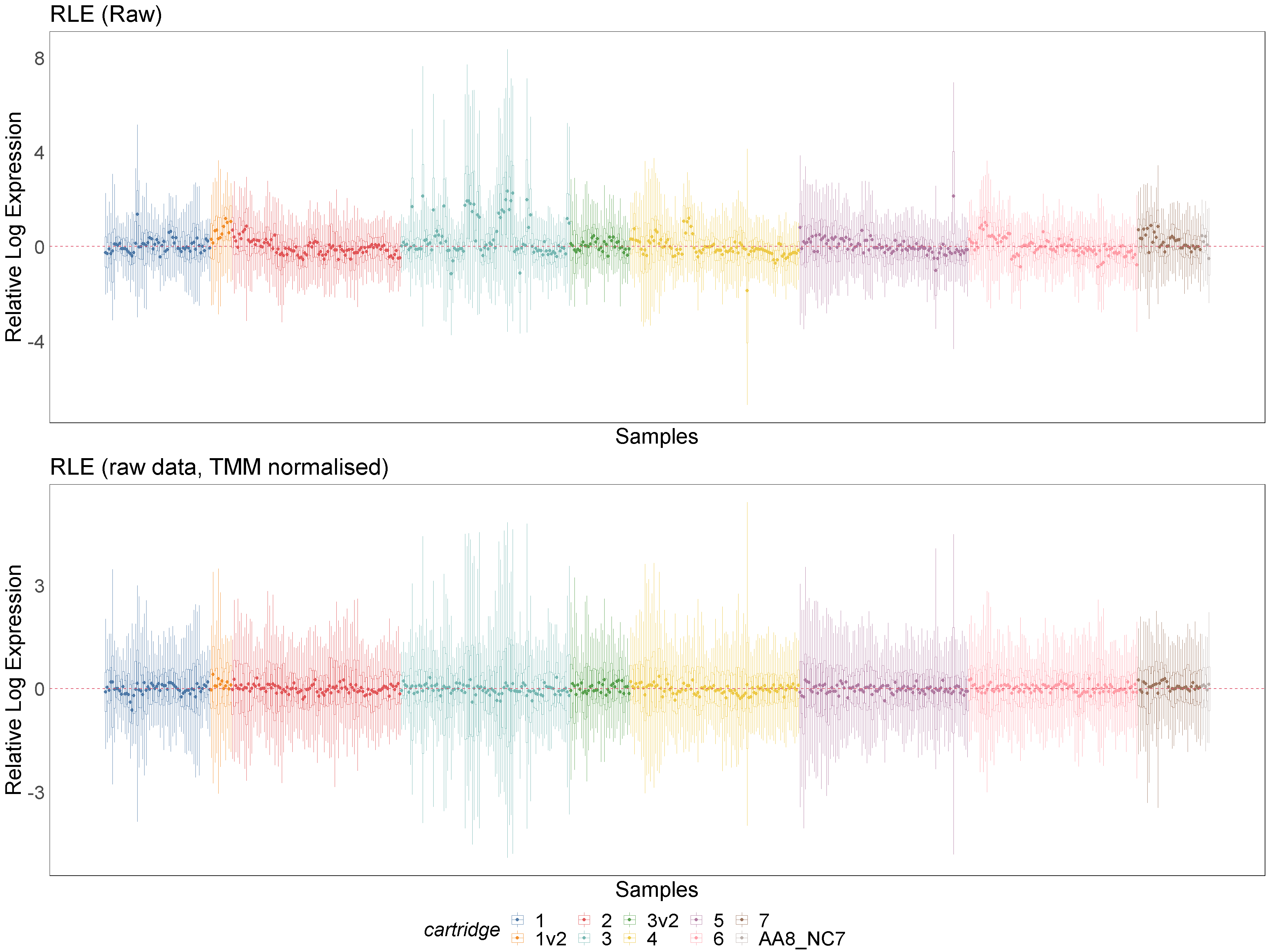


**Supplementary Figure 3: Analysis of the Relative Log Expression (RLE) of the raw and trimmed mean of M-values (TMM) spatial proteomics data.** Normalisation using TMM reduces data variability but not the variance.

**
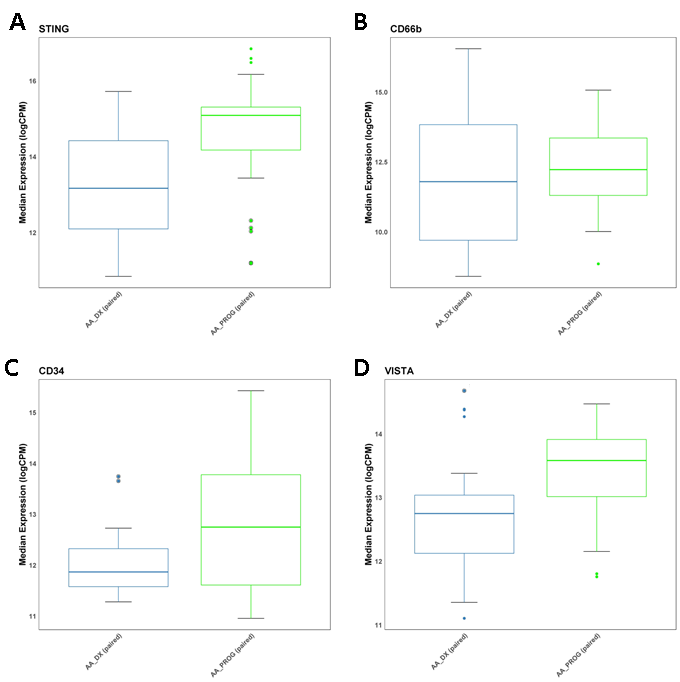
**

**Supplementary Figure 4: Comparison of AA_DX vs AA_PROG in paired samples does not identify any statistically significant changes in protein expression. (**A) STING, (B) CD66b, (C) CD34 and (D) VISTA.

**
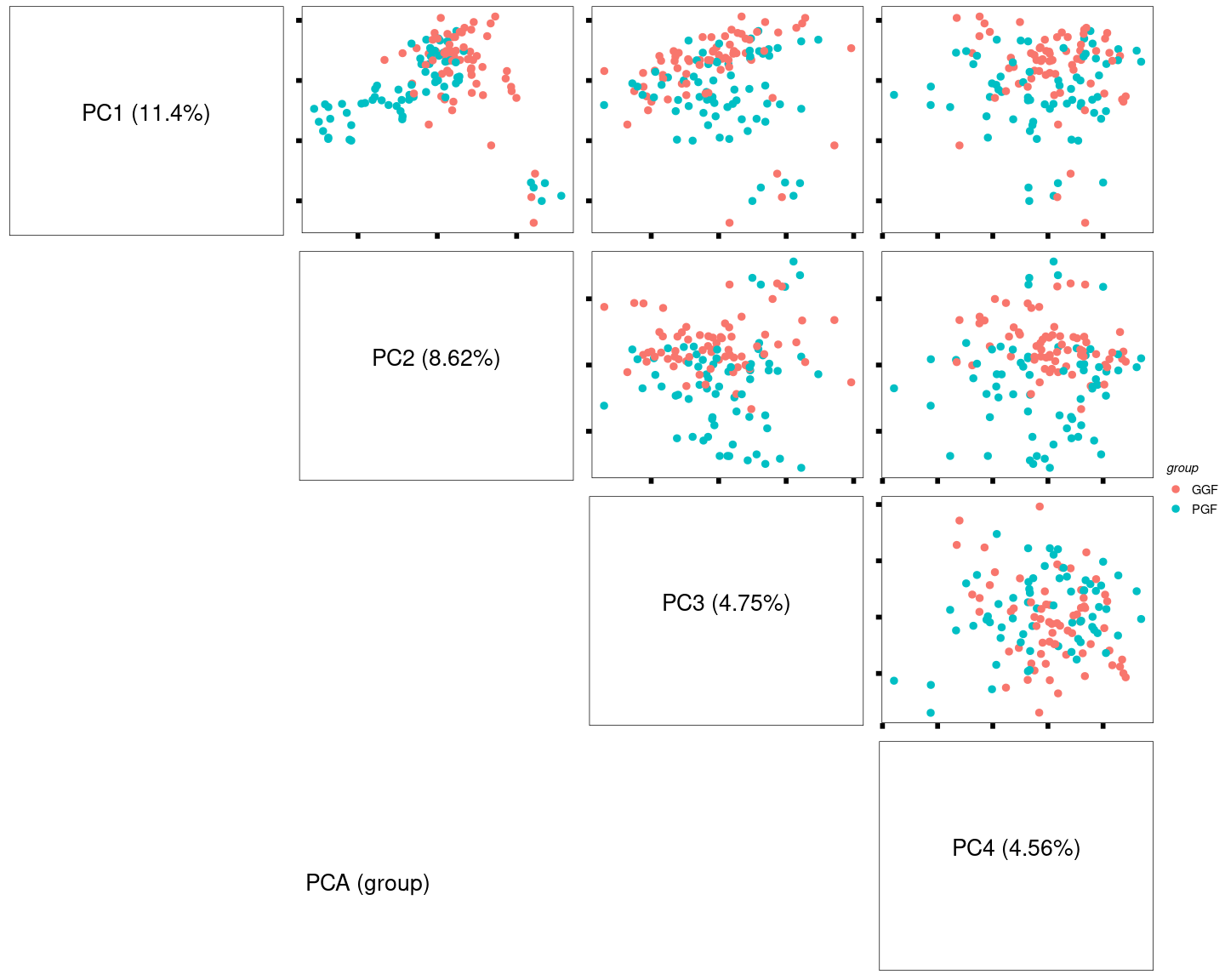
**

**Supplementary Figure 5: PCA of PGF and GGF demonstrates significant overlap between these groups.**

**
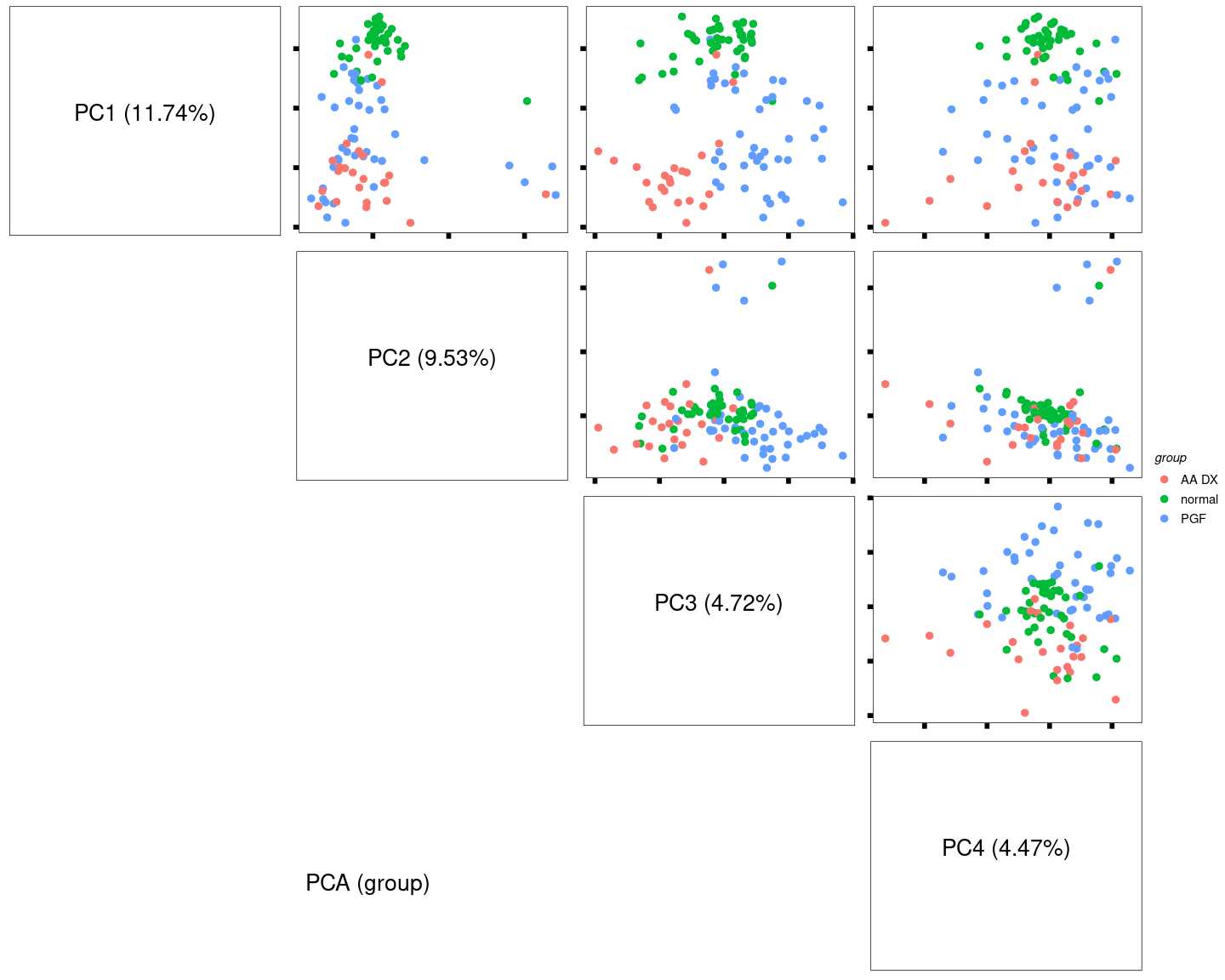
**

**Supplementary Figure 6: PCA of AA_DX, PGF and normal controls demonstrates separation based on disease group along PC1.**

**
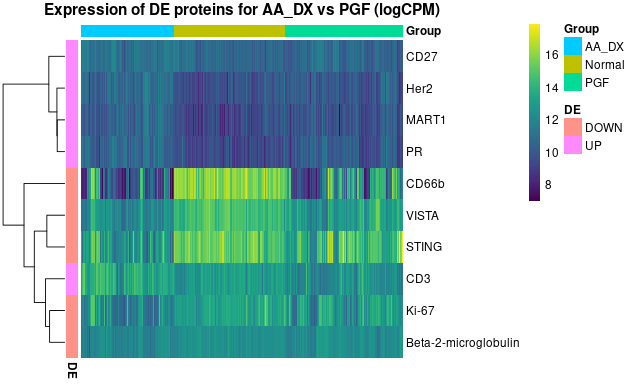
**

**Supplementary Figure 7: Heatmap of log normalised counts (logCPM) of differentially expressed (DE) proteins between AA_DX, PGF and normal controls.** For differential expression, expression was determined relative to PGF. As this analysis used pre-designed panels, it included markers that are not known to be expressed in the BM such as MART1, Her2 and NY-ESO-1. These markers were included in the statistical analysis but are not considered further for the dissection of tissue pathology.

**Supplementary Figure 8: Differences in PB immune subsets across AA, PGF, GGF and normal controls.** Flow cytometry analysis of PB samples from patients with AA (n=5), PGF (n=17), GGF (n=13) and normal controls (n=14) across (A) B cells, (B) NK cells, (C) CD4 and CD8 T cells, (D) Classical, Non-Classical and Intermediate Monocytes. *P<0.05, **P<0.01

**Supplementary Figure 9: STING and VISTA expression across T cell memory subsets.** Flow cytometry analysis of PB samples from patients with AA (n=5), PGF (n=17), GGF (n=13) and normal controls (n=14) across CD4 T cells for (A) STING and (B) VISTA expression and CD8 T cells for (C) STING and (D) VISTA expression across Naïve (CCR7^+^CD45RA^+^), Central Memory (CM; CCR7^+^CD45RA^-^), Effector Memory (EM; CCR7^-^CD45RA^-^) and Terminal Effector Memory expressing CD45RA (TEMRA; CCR7^-^CD45RA^+^) subsets. *P<0.05, **P<0.01, ***P<0.001, ****P<0.0001

**Supplementary Figure 10: Reduced absolute number of STING and VISTA CD8 (A-B) and CD4 (C-D) T cells in AA (n=4) and PGF (n=15) patients compared to GGF patients (n=13).**
